# Anesthetic Isoflurane Increases Phosphorylated Tau Levels Mediated by Caspase Activation and Aβ Generation

**DOI:** 10.1371/journal.pone.0039386

**Published:** 2012-06-20

**Authors:** Yuanlin Dong, Xu Wu, Zhipeng Xu, Yiying Zhang, Zhongcong Xie

**Affiliations:** 1 Geriatric Anesthesia Research Unit, Department of Anesthesia, Critical Care and Pain Medicine, Massachusetts General Hospital and Harvard Medical School, Charlestown, Massachusetts, United States of America; 2 Department of Forensic Pathology, Faculty of Forensic Medicine, China Medical University, Shenyang, People’s Republic of China; University of Nebraska Medical Center, United States of America

## Abstract

Anesthetic isoflurane has been shown to promote Alzheimer’s disease (AD) neuropathogenesis by inducing caspase activation and accumulation of β-amyloid (Aβ). Phosphorylation of tau protein is another important feature of AD neuropathogenesis. However, the effects of isoflurane on phosphorylated tau levels remain largely to be determined. We therefore set out to determine whether isoflurane can increase phosphorylated tau levels. 5 to 8 month-old wild-type and AD transgenic mice [B6.Cg-Tg (APPswe, PSEN1dE9)85Dbo/J] were treated with 1.4% isoflurane for two hours. The mice brain tissues were harvested at six, 12 and 24 hours after the anesthesia. For the *in vitro* studies, primary neurons from wild-type and the AD transgenic mice were exposed to 2% isoflurane for six hours, and were harvested at the end of anesthesia. The harvested brain tissues and neurons were subjected to Western blot analysis by which the levels of phosphorylated tau protein at Serine 262 (Tau-PS262) were determined. Here we show that the isoflurane anesthesia increased Tau-PS262 levels in brain tissues and primary neurons from the wild-type and AD transgenic mice. Moreover, the isoflurane anesthesia may induce a greater increase in Tau-PS262 levels in primary neurons and brain tissues from the AD transgenic mice. Finally, caspase activation inhibitor Z-VAD and Aβ generation inhibitor L-685,458 attenuated the isoflurane-induced increases in Tau-PS262 levels. In conclusion, clinically relevant isoflurane anesthesia increases phosphorylated tau levels, which may result from the isoflurane-induced caspase activation and Aβ generation. These findings will promote more studies to determine the effects of anesthetics on tau phosphorylation.

## Introduction

Alzheimer’s disease (AD) is an insidious and progressive neurodegenerative disorder. Currently, there is no cure for AD. However, if the onset of AD could be delayed by just one year in those who develop this mind-destroying ailment, the number of worldwide AD cases would be reduced by 12 million by 2050 (2nd Alzheimer’s Association International Conference on Prevention of Dementia in Washington, D.C., June, 2007). Therefore, it is important to identify any environmental factors which may promote AD development and AD neuropathogenesis.

An estimated 200 million patients worldwide undergo anesthesia and surgery each year. It has been reported that the age of AD onset is inversely related to cumulative exposure to anesthesia and surgery before age 50 [1], even though anesthesia and surgery may not increase the incidence of AD [2]. Several recent studies also reported that general anesthesia could be a risk factor for the development of AD [3], [4]. Finally, Tang et al. showed that anesthesia and surgery may lead to changes in cerebrospinal fluid biomakers consistent with AD [5]. However, other studies have suggested different findings [6], [7], [8]. Therefore, more population and basic studies defining the role of anesthesia in AD development and AD neuropathogenesis are needed [9].

AD neuropathogenesis includes intraneuronal neurofibrillary tangles that are composed of insoluble aggregates of hyperphosphorylated tau protein [10], [11], [12], reviewed in [13]. Tau protein is a microtubule associated protein abundantly found in neuronal axons. Hyperphosphorylated tau would dissociate from the microtubule and relocalize in the somatodendritic compartment, which would then destabilize the microtubule, causing neuronal dysfunction, neurodegeneration and ultimately functional deficits [14]. Recent studies have shown that anesthesia-induced hypothermia [15], [16], [17] and the commonly used anesthetic propofol [18] and sevoflurane [19] can induce phosphorylation of tau protein.

Isoflurane, one of the most commonly used inhalational anesthetics, has been shown to induce caspase activation and apoptosis, affect amyloid precursor protien (APP) processing, increase β-amyloid protein (Aβ) generation, enhance Aβ aggregation and impair learning and memory [20], [21], [22], [23], [24], [25], [26], [27], [28], [29], [30]. However, the effects of isoflurane on phosphorylated tau levels remain largely to be determined. In the present studies, we assessed the effects of isoflurane on phosphorylated tau levels and determined the extent to which these effects could result from the isoflurane-induced caspase activation and Aβ generation.

## Results

### Isoflurane Increased Phosphorylated Tau Levels in Brain Tissues of Wild-type (WT) and AD Transgenic (Tg) Mice

**Figure 1:**
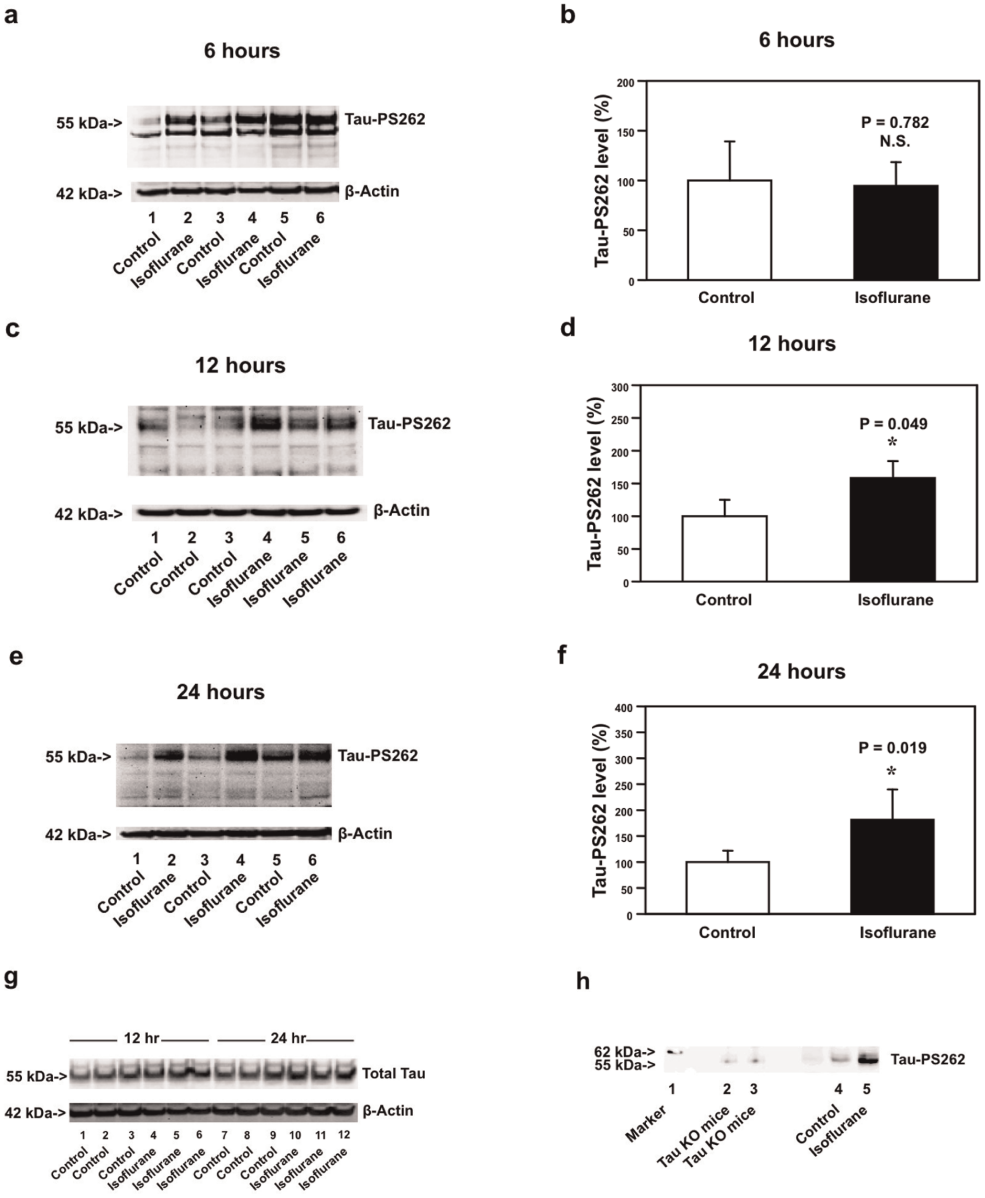
Isoflurane increases Tau-PS262 levels in brain tissues of WT mice. Isoflurane anesthesia (lanes 2, 4 and 6) does not increase PS262 levels as compared to the control condition (lanes 1, 3 and 5) in the brain tissues of WT mice at six hours after the isoflurane anesthesia. *b.* Quantification of the Western blot shows that isoflurane anesthesia (black bar, P = 0.782, N.S.) does not increase PS262 levels as compared to the control condition (white bar). *c.* Isoflurane anesthesia (lanes 4 to 6) increases Tau-PS262 levels as compared to the control condition (lanes 1 to 3) in the brain tissues of WT mice at 12 hours after the isoflurane anesthesia. *d.* Quantification of the Western blot shows that isoflurane anesthesia (black bar, *P = 0.049) increases Tau-PS262 levels as compared to the control condition (white bar). *e.* Isoflurane anesthesia (lanes 2, 4 and 6) increases Tau-PS262 levels as compared to the control condition (lanes 1, 3 and 5) in the brain tissues of WT mice at 24 hours after the isoflurane anesthesia. *f.* Quantification of the Western blot shows that isoflurane anesthesia (black bar, *P = 0.019) increases Tau-PS262 levels as compared to the control condition (white bar). *g.* Isoflurane anesthesia (lanes 4–6 and lanes 10–12) does not increase levels of total tau as compared to the control condition (lanes 1–3 and lanes 7–9) in the brain tissues of WT mice at 12 and 24 hours after the isoflurane anesthesia. *h.* Isoflurane anesthesia (lane 5) leads to a more visible band in the Western blot analysis (at about 55 kDa) as compared to control condition (lane 4) in WT mice brain tissues, whereas tau knockout (KO) mice brain tissues (lanes 2 and 3, the negative controls) do not show such band. We have averaged results from three to six independent experiments. WT, wild-type. N = 3–6.

We assessed the effects of isoflurane on phosphorylated tau levels in the brain tissues of 5–8 month-old WT and AD Tg mice [B6.Cg-Tg (APPswe, PSEN1dE9)85Dbo/J]. The WT mice were treated with 1.4% isoflurane for two hours. The brain tissues of the mice were harvested at six, 12 and 24 hours after the anesthesia, and were subjected to Western blot analysis. The immunoblotting of phosphorylated tau protein at serine 262 (Tau-PS262) showed that the isoflurane anesthesia did not increase the levels of Tau-PS262 at six hours after the anesthesia (Figure 1a). There was no significant difference in β-Actin levels between the control condition and isoflurane-treated mice. Quantification of the Western blot, based on the ratio of Tau-PS262 and β-Actin, showed that the isoflurane treatment did not increase Tau-PS262 levels six hours post-anesthesia (Figure 1b). The isoflurane anesthesia increased the levels of Tau-PS262 at 12 hours after the anesthesia (Figure 1c). The quantification of the Western blot, based on the ratio of Tau-PS262 and β-Actin, showed that the isoflurane anesthesia led to increases in the levels of Tau-PS262 in the mice brain tissues at 12 hours after the anesthesia: 100% versus 161%, *P = 0.049 (Figure 1d). The isoflurane anesthesia increased the levels of Tau-PS262 in mice brain tissues at 24 hours after the anesthesia (Figure 1e and Figure 1f): 100% versus 177%, *P = 0.019. The isoflurane anesthesia did not increase the levels of total tau in WT mice brain tissues (Figure 1g). Finally, we used tau knockout mice brain tissues as negative controls in our studies and found that isoflurane anesthesia led to a more visible band in the Western blot analysis (at about 55 kDa) as compared to control condition in WT mice brain tissues, whereas tau knockout mice brain tissues did not show such band (Figure 1h). These data indicated that the bands (55 kDa) observed following the isoflurane anesthesia in current experiments were phosphorylated tau. Taken together, these results suggest that isoflurane anesthesia may induce tau phosphorylation in WT mice brain tissues, as evidenced that the isoflurane anesthesia increased the levels of phosphorylated tau, but not total tau.

**Figure 2:**
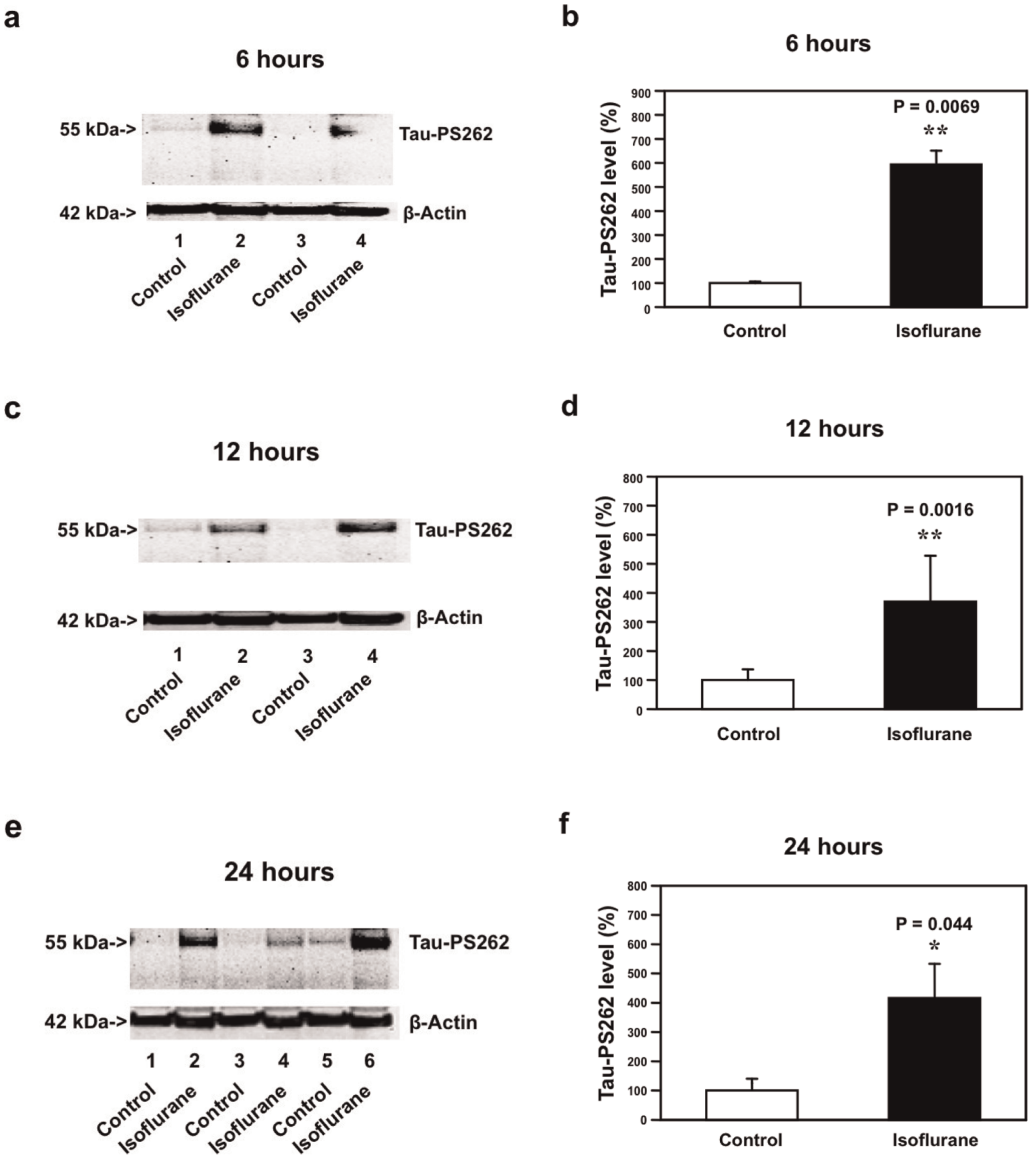
Isoflurane increases Tau-PS262 levels in brain tissues of AD Tg mice. *a.* Isoflurane anesthesia (lanes 2 and 4) increases Tau-PS262 levels as compared to the control condition (lanes 1 and 3) in the brain tissues of AD Tg mice at six hours after the isoflurane anesthesia. *b.* Quantification of the Western blot shows that isoflurane anesthesia (black bar, **P = 0.0069) increases Tau-PS262 levels as compared to the control condition (white bar). *c.* Isoflurane anesthesia (lanes 2 and 4) increases Tau-PS262 levels as compared to the control condition (lanes 1 and 3) in brain tissues of AD Tg mice at 12 hours after the isoflurane anesthesia. *d.* Quantification of the Western blot shows that isoflurane anesthesia (black bar, **P = 0.0016) increases Tau-PS262 levels as compared to the control condition (white bar). *e.* Isoflurane anesthesia (lanes 2, 4 and 6) increases Tau-PS262 levels as compared to the control condition (lanes 1, 3 and 5) in brain tissues of AD Tg mice at 24 hours after the isoflurane anesthesia. *f.* Quantification of the Western blot shows that isoflurane anesthesia (black bar, *P = 0.044) increases Tau-PS262 levels as compared to the control condition (white bar). We have averaged results from three independent experiments. AD, Alzheimer’s disease, Tg, transgenic. N = 3.

Given the findings that the isoflurane anesthesia may increase Tau-PS262 levels in brain tissues of WT mice, next, we assessed whether elevated Aβ levels could potentiate the isoflurane-induced increases in Tau-PS262 levels. Five to 8 month-old AD Tg mice [B6.Cg-Tg(APPswe, PSEN1dE9) 85Dbo/J mice] were treated with 1.4% isoflurane for two hours. The brain tissues of these mice were harvested at six, 12 and 24 hours after the anesthesia, and were subjected to Western blot analysis. The isoflurane anesthesia increased Tau-PS262 levels in mice brain tissues at six hours after the anesthesia: 100% versus 588%, **P = 0.00687 (Figure 2a and 2b). Similarly, the isoflurane anesthesia led to increases in the levels of Tau-PS262 in the AD Tg mice brain tissues at 12 hours: 100% versus 377%, **P = 0.0016 (Figure 2c and Figure 2d); and 24 hours after the anesthesia: 100% versus 401%, *P = 0.044 (Figure 2e and Figure 2f). Taken together, these results suggest that isoflurane can increase phosphorylated tau levels in the brain tissues of both WT and AD Tg mice. Moreover, these findings suggest that isoflurane may induce a greater increase in phosphorylated tau levels in AD Tg mice brain than WT mice brain (Figure 1 versus Figure 2), which is consistent with the findings that Aβ can potentiate tau phosphorylation [31].

### Isoflurane Increased Phosphorylated Tau Levels in WT and AD Tg mice Primary Neurons

Given that isoflurane may induce a greater increase in Tau-PS262 levels in AD Tg mice brain tissues than in WT mice brain tissues, next we assessed the effects of isoflurane on Tau-PS262 levels in primary neurons from WT and AD Tg mice and further asked whether elevated Aβ level may potentiate the isoflurane-induced increase in phosphorylated tau levels.

**Figure 3:**
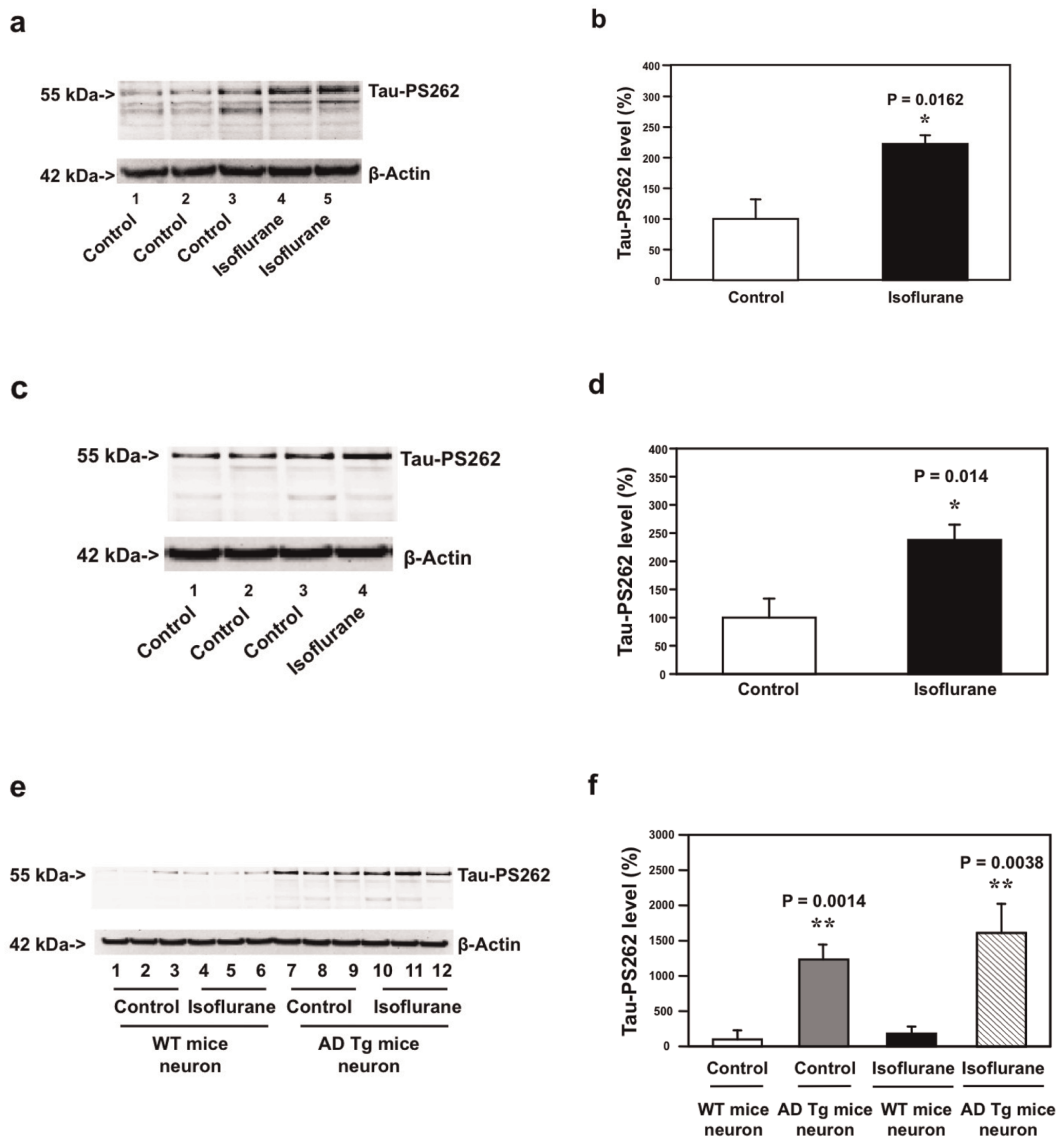
Isoflurane increases Tau-PS262 levels in WT and AD Tg mice primary neurons. *a.* Isoflurane treatment (lanes 4 and 5) increases Tau-PS262 levels as compared to the control condition (lanes 1 to 3) in WT mice primary neurons. *b.* Quantification of the Western blot shows that isoflurane treatment (black bar, *P = 0.0162) increases Tau-PS262 levels as compared to the control condition (white bar). *c.* Isoflurane treatment (lane 4) increases Tau-PS262 levels as compared to the control condition (lanes 1 to 3) in AD Tg mice primary neurons. *d.* Quantification of the Western blot shows that isoflurane treatment (black bar, *P = 0.014) increases Tau-PS262 levels as compared to the control condition (white bar) in AD Tg mice primary neurons. *e.* The baseline levels of Tau-PS262 levels in AD Tg mice primary neurons (lanes 7 to 9) are higher than those in WT mice primary neurons (lanes 1 to 3); and isoflurane treatment (lanes 10 to 12) induces a greater increase in Tau-PS262 levels in AD Tg mice primary neurons than in WT mice primary neurons (lanes 4 to 6). *f.* Quantification of the Western blot shows that there is a higher baseline Tau-PS262 levels in AD Tg mice primary neurons (gray bar) than WT mice primary neurons (white bar): **P = 0.0014; and isoflurane induces a greater increase in Tau-PS262 levels in AD Tg mice primary neurons (net bar) than in WT mice primary neurons (black bar): **P = 0.0038. We have averaged results from 6 to 12 independent experiments. WT, wild-type, AD, Alzheimer’s disease, Tg, transgenic. N = 6–12.

WT mice primary neurons were treated with 2% isoflurane for six hours. The neurons were harvested at the end of the experiments and subjected to Western blot analysis. The immunoblotting of Tau-PS262 showed that the isoflurane treatment increased Tau-PS262 levels (Figure 3a). There was no significant difference in β-Actin levels between the control condition and isoflurane-treated neurons. Quantification of the Western blot, based on the ratio of Tau-PS262 and β-Actin, showed that the isoflurane treatment led to increases in Tau-PS262 levels: 100% versus 234%, *P = 0.0162 (Figure 3b).

Next, we assessed the effects of isoflurane on Tau-PS262 levels in AD Tg mice primary neurons. Immunoblotting of Tau-PS262 showed that the isoflurane treatment increased the levels of Tau-PS262 in AD Tg mice primary neurons (Figure 3c). There is no significant difference in β-Actin levels between the control condition and isoflurane-treated neurons. Quantification of the Western blot showed that the isoflurane treatment led to increases in Tau-PS262 levels: 100% versus 243%, *P = 0.014 (Figure 3d).

The side by side comparison between WT mice primary neurons and AD Tg mice primary neurons showed that there were higher baseline levels of Tau-PS262 in AD Tg mice primary neurons than in WT mice primary neurons: lanes 1 to 3 versus lanes 7 to 9 (Figure 3e), 100% versus 1,207%, **P = 0.0014 (Figure 3f). The isoflurane anesthesia also led to a greater increase in Tau-PS262 levels in AD Tg mice primary neurons than in WT mice primary neurons: lanes 4 to 6 versus lanes 10 to 12 (Figure 3e), 207% versus 1,677%, **P = 0.0038 (Figure 3f). Finally, we were able to show that there were higher Aβ levels in the primary neurons from AD Tg mice than those in the primary neurons from WT mice (Table 1): 100 versus 62 pmol/ml, *P = 0.019. Taken together, these findings suggest that isoflurane may induce a greater increase in phosphorylated tau levels when there are higher Aβ levels, e.g., in AD Tg mice.

**Table 1:** Aβ42 levels in the primary neurons from WT and AD Tg mice.

	Primary neurons from WT mice	Primary neurons from AD Tg mice
Extracellular Aβ42 (pmol/ml)	62±2.8	100±17.0 (*P = 0.019)

Extracellular Aβ42 levels are higher in the primary neurons from AD Tg mice than those in the primary neurons from WT mice. N = 6.

Aβ, β-amyloid protein; WT, wild-type; AD, Alzheimer’s disease; Tg, transgenic.

### Cardobenzoxy-valyl-alanyl-aspartyl-(O-methyl)-fluoromethylketone (Z-VAD) and L-685,458 Attenuated the Isoflurane-induced Increase in Tau-PS262 levels in WT and AD Tg mice Primary Neurons

Our previous studies have shown that isoflurane may increase Aβ generation via the effects of isoflurane on inducing caspase activation and apoptosis [27]. Z-VAD is a caspase activation inhibitor and L-685,458 is an Aβ generation inhibitor (γ-secretase inhibitor). Both Z-VAD and L-685,458 have been shown to attenuate the isoflurane-induced caspase-3 activation and Aβ accumulation, respectively [27]. Our current studies suggest that Aβ may potentiate the isoflurane-induced increase in phosphorylated tau levels (Figures 1, 2 and 3). We therefore assessed whether reductions in caspase activation and Aβ generation by Z-VAD and L-685,458, respectively, could attenuate the isoflurane-induced increase in phosphorylated tau levels in WT and AD Tg mice primary neurons.

**Figure 4:**
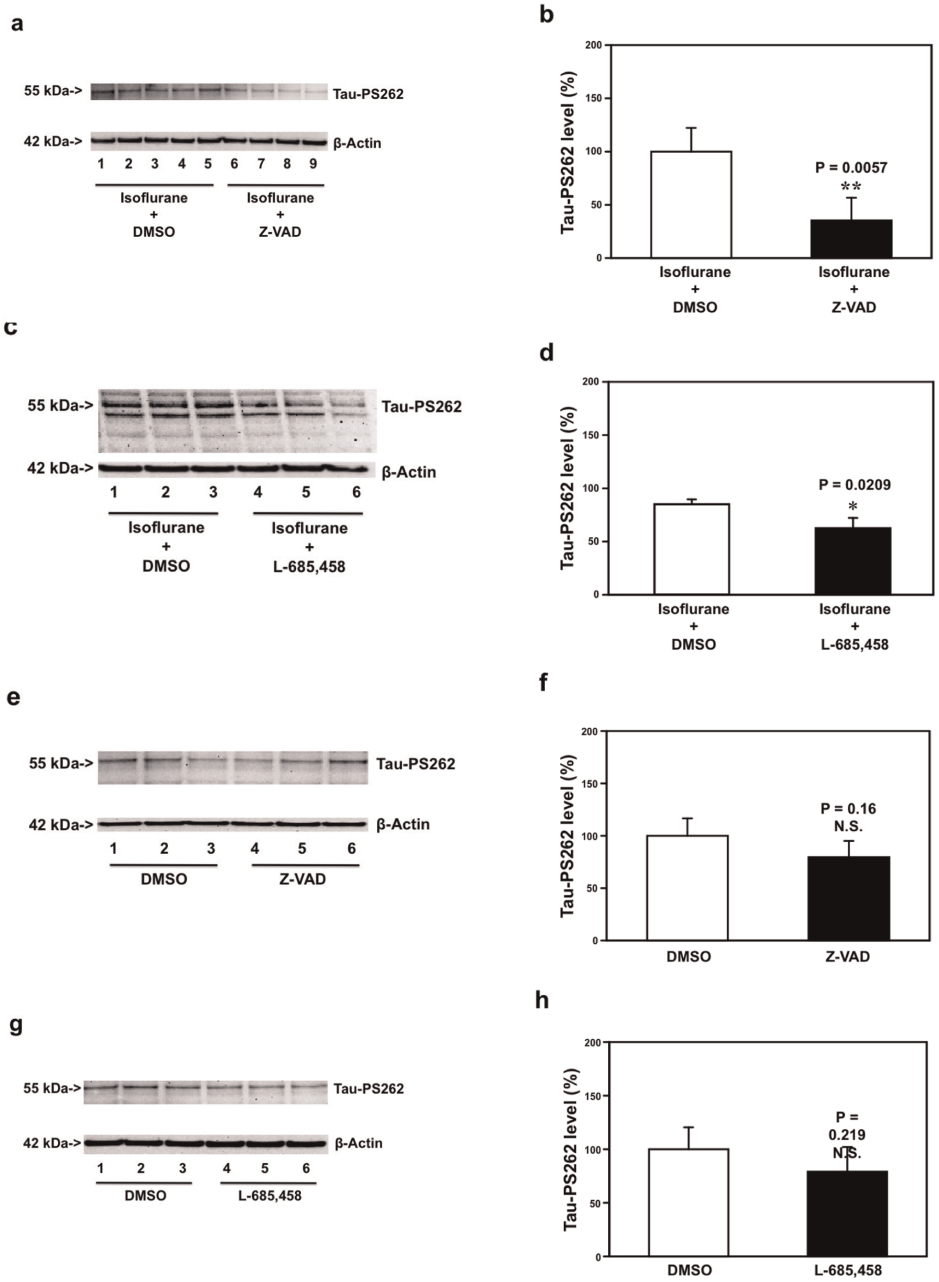
Z-VAD and L-685,458 attenuate the isoflurane-induced increase in Tau-PS262 levels in WT mice primary neurons. *a.* Treatment with isoflurane plus Z-VAD (lanes 6 to 9) leads to reductions in Tau-PS262 levels as compared to treatment with isoflurane plus DMSO (lanes 1 to 5) in WT mice primary neurons. *b.* Quantification of the Western blot shows that treatment with isoflurane plus Z-VAD (black bar, **P = 0.0057) leads to a reduction in Tau-PS262 levels as compared to isoflurane plus DMSO (white bar). *c.* Treatment with isoflurane plus L-685,458 (lanes 4 to 6) leads to reductions in Tau-PS262 levels as compared to treatment with isoflurane plus DMSO (lanes 1 to 3) in WT mice primary neurons. *d.* Quantification of the Western blot shows that treatment with isoflurane plus L-685,458 (black bar, *P = 0.0209) leads to reductions in Tau-PS262 levels as compared to isoflurane plus DMSO (white bar). *e.* Z-VAD (lanes 4 to 6) alone does not significantly affect Tau-PS262 levels as compared to DMSO (lanes 1 to 3) in WT mice primary neurons. *f.* Quantification of the Western blot shows that Z-VAD (black bar, P = 0.16, N.S.) does not significantly alter Tau-PS262 levels as compared to DMSO (white bar) in WT mice primary neurons. *g.* L-685,458 (lanes 4 to 6) alone does not significantly affect Tau-PS262 levels as compared to DMSO (lanes 1 to 3) in WT mice primary neurons. *h.* Quantification of the Western blot shows that L-685,458 (black bar, P = 0.219, N.S.) does not significantly alter Tau-PS262 levels as compared to DMSO (white bar) in WT mice primary neurons. We have averaged the results from 6 to 12 independent experiments. WT, wild-type; Z-VAD, Cardobenzoxy-valyl-alanyl-aspartyl-(O-methyl)-fluoromethylketone; DMSO, Dimethyl sulfoxide. N = 6−12.

Treatment with isoflurane plus Z-VAD led to reductions in the levels of Tau-PS262 as compared to the treatment with isoflurane plus dimethyl sulfoxide (DMSO, the vehicle of Z-VAD in the experiments) (Figure 4a). The quantification of the Western blot showed that the treatment with isoflurane plus Z-VAD (black bar, Figure 4b) led to reductions in the levels of Tau-PS262 as compared to the treatment with isoflurane plus DMSO (white bar, Figure 4b): 100% versus 43%, **P = 0.0057. The treatment with isoflurane plus L-685,458 (lanes 4 to 6 in Figure 4c; black bar in Figure 4d) led to reductions in the levels of Tau-PS262 as compared to the treatment with isoflurane plus DMSO (the vehicle of L-685,458 in the experiments) (lanes 1 to 3 in Figure 4c, white bar in Figure 4d): 100% versus 69%, *P = 0.0209. Moreover, treatment with Z-VAD (Figure 4e and 4f, P = 0.16, N.S.) or L-685,458 (Figure 4g and 4h, P = 0.219, N.S.) alone did not significantly affect Tau-PS262 levels as compared to DMSO in primary neurons from WT mice. These results suggest that whereas Z-VAD or L-685,458 alone does not significantly affect phosphorylated tau levels, Z-VAD and L-685,458 may attenuate the isoflurane-induced increase in phosphorylated tau levels in primary neurons from WT mice.

**Figure 5:**
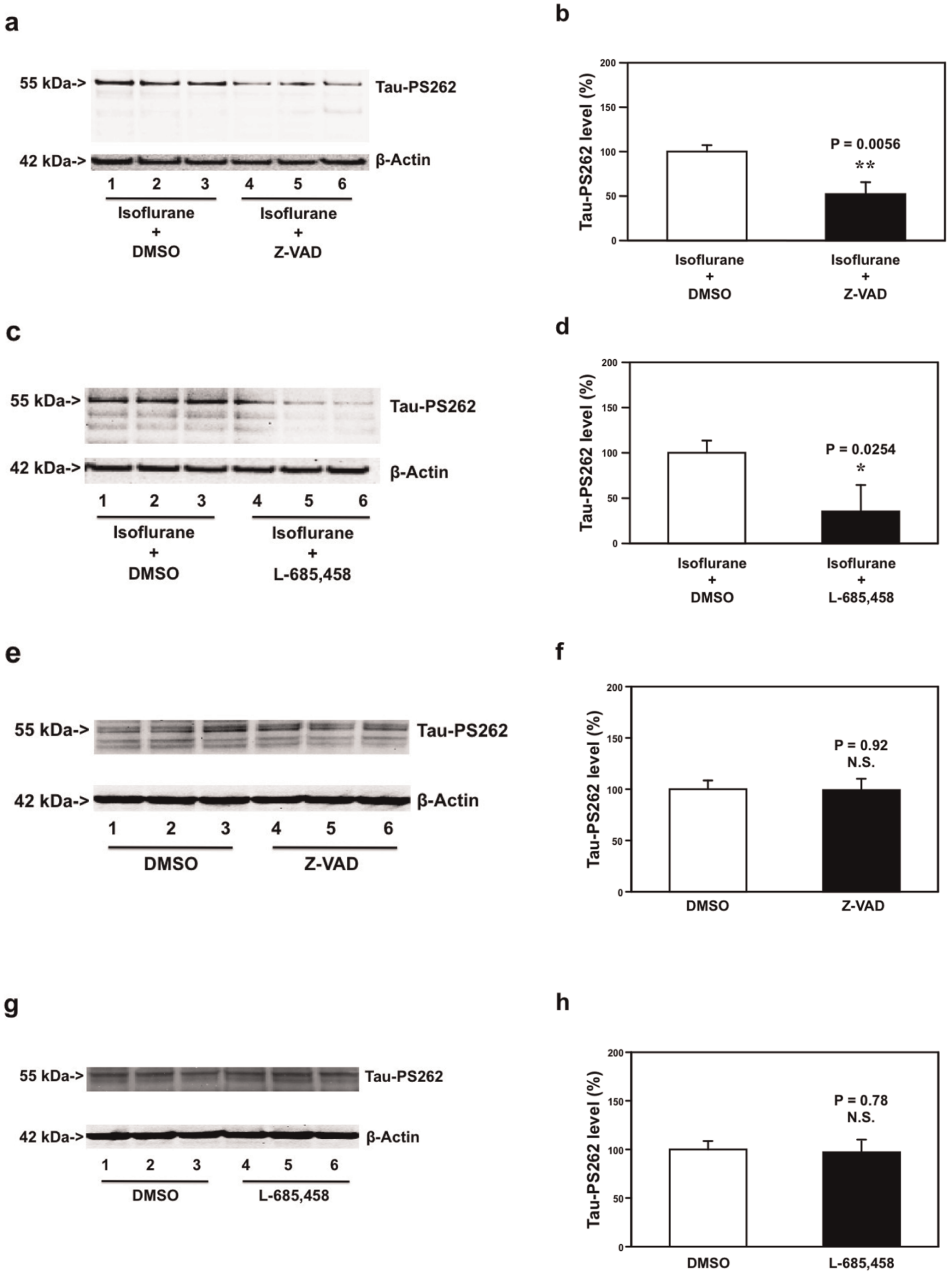
Z-VAD and L-685,458 attenuate the isoflurane-induced increase in Tau-PS262 levels in AD Tg mice primary neurons. *a.* Treatment with isoflurane plus Z-VAD (lanes 4 to 6) leads to reductions in Tau-PS262 levels as compared to treatment with isoflurane plus DMSO (lanes 1 to 3) in AD Tg mice primary neurons. *b.* Quantification of the Western blot shows that treatment with isoflurane plus Z-VAD (black bar, **P = 0.0056) leads to reductions in Tau-PS262 levels as compared to isoflurane plus DMSO (white bar). *c.* Treatment with isoflurane plus L-685,458 (lanes 4 to 6) leads to reductions in Tau-PS262 levels as compared to treatment with isoflurane plus DMSO (lanes 1 to 3) in AD Tg mice primary neurons. *d.* Quantification of the Western blot shows that treatment with isoflurane plus L-685,458 (black bar, *P = 0.0254) leads to reductions in Tau-PS262 levels as compared to isoflurane plus DMSO (white bar). *e.* Z-VAD (lanes 4 to 6) alone does not significantly affect Tau-PS262 levels as compared to DMSO (lanes 1 to 3) in AD Tg mice primary neurons. *f.* Quantification of the Western blot shows that Z-VAD (black bar, P = 0.92, N.S.) does not significantly alter Tau-PS262 levels as compared to DMSO (white bar) in AD Tg mice primary neurons. *g.* L-685,458 (lanes 4 to 6) alone does not significantly affect Tau-PS262 levels as compared to DMSO (lanes 1 to 3) in AD Tg mice primary neurons. *h.* Quantification of the Western blot shows that L-685,458 (black bar, P = 0.78, N.S.) does not significantly alter Tau-PS262 levels as compared to DMSO (white bar) in AD Tg mice primary neurons. We have averaged the results from 6 to 12 independent experiments. AD, Alzheimer’s disease; Tg, transgenic; Z-VAD, Cardobenzoxy-valyl-alanyl-aspartyl-(O-methyl)-fluoromethylketone; DMSO, dimethyl sulfoxide. N = 6−12.

Finally, treatment with isoflurane plus Z-VAD led to reductions in the levels of Tau-PS262 as compared to the treatment with isoflurane plus DMSO (Figure 5a). The quantification of the Western blot showed that the treatment with isoflurane plus Z-VAD (black bar, Figure 5b) led to reductions in the levels of Tau-PS262 as compared to the treatment with isoflurane plus DMSO (white bar, Figure 5b): 100% versus 53%, **P = 0.0056. The treatment with isoflurane plus L-685,458 (lanes 4 to 6 in Figure 5c; black bar in Figure 5d) led to reductions in the levels of Tau-PS262 as compared to the treatment with isoflurane plus DMSO (lanes 1 to 3 in Figure 5c, white bar in Figure 5d): 100% versus 39%, *P = 0.0254. The treatment with Z-VAD or L-685,458 alone did not significantly alter the levels of Tau-PS262 as compared to DMSO (Figure 5e, 5f, 5g and 5h). These results suggest that both Z-VAD and L-685,458 may attenuate the isoflurane-induced increase in phosphorylated tau levels in primary neurons from AD Tg mice. Collectively, these findings suggest that isoflurane may induce caspase activation, apoptosis and Aβ accumulation, which then lead to increase in phosphorylated tau levels.

## Discussion

The commonly used inhalation anesthetic isoflurane has been shown to promote AD neuropathogenesis by inducing caspase activation, apoptosis, Aβ accumulation, neuroinflammation and mitochondrial dysfunction [20], [21], [22], [23], [24], [25], [26], [27], [28], [30], [32], [33], [34]. Tau phosphorylation is part of AD neuropathogenesis, we therefore assessed the effects of isoflurane on phosphorylated tau levels.

We first found that clinically relevant isoflurane anesthesia increased levels of phosphorylated tau protein at Serine 262 (Tau-PS262) in brain tissues of WT (Figure 1) and AD Tg mice (Figure 2) at six, 12 and 24 hours after the anesthesia. The isoflurane anesthesia did not significantly affect levels of total tau (Figure 1g), Tau-PS199, and Tau-PS422 (data not shown) in brain tissues of WT mice. The findings that the isoflurane anesthesia increased levels of Tau-PS262 but not total tau suggest that isoflurane may induce tau phosphorylation in brain tissues of WT mice. We did not assess effects of isoflurane anesthesia on total tau levels in the brain tissues of AD Tg mice or in primary neurons from both WT and AD Tg mice because the objective of these studies is to determine the role of caspase activation and Aβ generation in the isoflurane-induced increase in phosphorylated tau levels.

Furthermore, we found that the isoflurane anesthesia led to greater increases in Tau-PS262 levels in AD Tg mice brain tissues than in WT mice brain tissues. There are higher Aβ levels in the AD Tg mice [B6.Cg-Tg(APPswe, PSEN1dE9) 85Dbo/J mice] brain tissues than in WT mice brain tissues [35], therefore these results suggest that Aβ may potentiate the isoflurane-induced increase in phosphorylated tau levels. We did not measure the effects of isoflurane on total tau levels in brain tissues of AD Tg mice because the isoflurane anesthesia did not increase total tau levels in brain tissues of WT mice and we only wanted to compare the effects of isoflurane on brain phosphorylated tau (e.g., Tau-PS262) levels between WT and the AD Tg mice.

Next, we found that a clinically relevant concentration of isoflurane increased levels of Tau-PS262 in primary neurons from WT and AD Tg mice primary neurons. Moreover, there were higher levels of Tau-PS262 in the AD Tg mice primary neurons as compared to WT mice primary neurons following control condition or isoflurane treatment (Figure 3). Given that there are elevated Aβ levels in AD Tg mice primary neurons as compared to WT mice primary neurons (Table 1), these results suggest that isoflurane can increase phosphorylated tau levels *in vitro* in primary neurons and moreover isoflurane may induce a greater increase in phosphorylated tau levels in the condition of higher Aβ levels.

We assessed the effects of isoflurane on Tau-PS262 levels because phosphorylation at the sites including S262 has been shown to prevent tau from binding and stabilizing microtubules [36], [37], [38], [39]. In addition, we also determined the effects of isoflurane on the levels of Tau-PS199 and Tau-PS422. Even these sites may not be within the microtubule binding region, the phosphorylation of these site in tau have been shown to be associated with severity of neuronal cytopathology in AD [40].

Finally, we were able to show that both caspase activation inhibitor Z-VAD and Aβ generation inhibitor L-685,458 (γ-secretase inhibitor) attenuated the isoflurane-induced increase in phosphorylated tau levels in WT (Figure 4) and AD Tg (Figure 5) mice primary neurons. These results suggest that the isoflurane-induced increase in phosphorylated tau levels may result from the isoflurane-induced caspase activation and Aβ generation. Our previous studies have shown that isoflurane-induced caspase-3 activation can cause Aβ generation [27]. Collectively, we have postulated that isoflurane induces caspase-3 activation, which increases Aβ generation, and the generated Aβ then leads to increases in phosphorylated tau levels.

Small and Duff have described the hypothesized “dual pathway model” and “serial model” of Aβ and tau causality [13]. In the serial model, it has been postulated that an insult (e.g., environmental factor) can increase Aβ levels, which then induce tau phosphorylation, leading to synaptic loss and dementia. In the dual pathway model, it has been hypothesized that an insult can induce Aβ elevation and tau phosphorylation simultaneously, which then separately lead to synaptic loss and dementia [13]. The findings from the current studies that isoflurane may increase phosphorylated tau levels via the effects of isoflurane on increasing Aβ levels support the “serial model”. Moreover, the findings from the current studies are consistent with the results from a recent study that Aβ isolated from the AD patient cortex can directly induce tau phosphorylation and neuritic degeneration [31]. Future studies may include assessing whether isoflurane can induce an Aβ- and tau-dependent synaptic loss and impairment of learning and memory to further illustrate the effects of isoflurane on AD neuropathogenesis. Finally, future studies should also include assessing whether the isoflurane-induced tau phosphorylation can induce caspase activation and Aβ accumulation, forming a vicious cycle of tau phosphorylation and caspase activation/Aβ generation.

Planel et al. have shown that isoflurane does not induce tau hyperphosphorylation in mice [15], [17], which is different from the findings of the current studies. The reason for these different findings remains largely to be determined. The isoflurane anesthesia in the studies by Planel et al. [17] consisted of four exposures of 1.3% isoflurane in 30% O2 for four hours, whereas the isoflurane anesthesia in the current studies was 1.4% isoflurane in 100% O2 for two hours. In addition, tau hyperphosphorylation was determined in mouse brain tissue at two hours or one week after the isoflurane anesthesia in the studies by Planel et al, while phosphorylated tau levels were determined in mouse brain tissue at six, 12 and 24 hours after the isoflurane anesthesia in the current studies. Furthermore, the isoflurane anesthesia in the current studies indeed did not increase phosphorylated tau levels at six hours after the anesthesia in the brain tissues of WT mice. Taken together, it is conceivable that different anesthesia and different mouse brain tissue harvest times may account for the different findings between the current studies and the studies by Planel et al. [17]. Further studies to test this hypothesis are warranted.

It is clinically difficult to prove and disprove the relationship between anesthesia and AD, and prospective clinical studies may take many years to conduct and analyze. Moreover, clinical studies have inevitable limitations owing to the characteristics of the diversity and potential confounding factors e.g., age, outcome measure, time of investigation, and kind of anesthesia/surgery. In contrast, animal studies of anesthesia neurotoxicity have less diversity and fewer confounding factors because animal populations are more homogeneous and all undergo standardized procedures and outcome measures in such studies. Therefore, while it is important to continue clinical studies, there is also a need to perform animal studies to investigate the potential neurotoxicity of anesthesia in AD neuropathogenesis. Moreover, the animal studies will allow us to establish a mechanistic hypothesis, vulnerable windows, less provocative anesthetics, and potential treatments, which may facilitate and guide more focused, uniform, and targeted clinical research. The current research will likely promote more studies to determine anesthesia neurotoxicity and the effects of anesthesia, surgery and other perioperative factors on AD neuropathogenesis.

The studies have several limitations. First, we only determined the effects of isoflurane anesthesia on the levels of total tau, Tau-PS262, Tau-PS199 and Tau-PS422 and found that isoflurane increased levels of Tau-PS262 but not total tau, Tau-PS199, and Tau-PS422 (data not shown) in brain tissues of WT mice. The future studies will include the systematic assessment of isoflurane’s effects on tau phosphorylation at other sites [e.g., AT8 (Ser202/Thr205), CP13 (Ser202), and PHF-1 (Ser396/Ser404) [18]] as well as the pathological and functional relevance of the isoflurane-induced tau phosphorylation. Second, we only determined the effects of isoflurane anesthesia on total tau levels in brain tissues of WT mice, but not in brain tissue of AD Tg mice or primary neurons. We thus may not conclude that isoflurane induces tau phosphorylation in AD Tg mice or primary neurons. However, the studies in AD Tg mice and primary neurons primarily aimed to determine the role of Aβ in the isoflurane-induced increase in phosphorylated tau levels. Nevertheless, the current studies have established a system and illustrated that isoflurane may induce tau phosphorylation in brain, and that the isoflurane-induced increase in phosphorylated tau levels could result from the isoflurane-induced caspase activation and Aβ generation. These findings will lead to more systematical studies of anesthetics on tau phosphorylation, including assessment of dose- and time-dependent effects of anesthetics on levels of both phosphorylated tau and total tau. Finally, our previous studies have shown that isoflurane, but not desflurane, can induce caspase activation and Aβ generation [25], [33], [34], [41], and propofol can attenuate the isoflurane-induced caspase activation [42], thus, the future studies will also include the comparison of the effects of isoflurane and other anesthetics, e.g., sevoflurane, desflurane and propofol, on tau phosphorylation. Isoflurane may induce learning and memory impairment independent of the isoflurane-induced Aβ accumulation and tau phosphorylation [30], [43]. Thus, future studies will include determining the cause-effect relationship of the anesthetics-induced tau phosphorylation and neurobehavioral deficits by assessing whether anti-tau treatment(s) can attenuate the isoflurane-induced neurobehavioral deficits.

### Conclusion

In conclusion, we have established a system to determine the effects of anesthetic on tau phosphorylation, the feature of AD neuropathogenesis, and underlying mechanisms *in vivo* and in primary neurons. We have found that a clinically relevant concentration of isoflurane can increase phosphorylated tau levels in primary neurons and the brain tissues of both WT and AD Tg mice. Aβ may potentiate the isoflurane-induced increase in phosphorylated tau levels. Finally, the isoflurane-induced increases in phosphorylated tau levels may result from the isoflurane-induced caspase activation and Aβ generation. These studies would promote more research to investigate the effects of anesthesia on tau phosphorylation and other AD neuropathogenesis, which may include assessing the effects of different anesthetics (e.g., isoflurane versus desflurane) on the levels of phosphorylated tau at different epitopes, total tau, tau-associated kinases and phosphatases in both WT and AD Tg mice, as well as the functional relevance (e.g., learning and memory impairment). Future studies may also include employing different methodology, e.g., immunohistochemistry, to define anesthetic-induced tau phosphorylation in different brain regions, e.g., the hippocampus versus the cortex. Ultimately, all of these efforts should facilitate the design of safer anesthetics and provision of better anesthesia care to patients, especially senior patients, who are particularly susceptible to developing postoperative cognitive dysfunction and AD.

## Methods

### Mice Anesthesia

Mice were used to assess the potential *in vivo* effects of isoflurane on levels of phosphorylated tau protein. The protocol was approved by the Massachusetts General Hospital (Boston, Massachusetts) Standing Committee on the Use of Animals in Research and Teaching. Wild-type (WT) mice (C57BL/6 mice) and AD transgenic (Tg) mice [B6.Cg-Tg (APPswe, PSEN1dE9)85Dbo/J mice] (Jackson Laboratory, Bar Harbor, ME) (donating from lab of David Borchelt, McKnight Brain Institute, University of Florida, Gainesville, FL, U.S.A.) at 5–8 months old were randomized by weight and gender into either an experimental group that received 1.4% isoflurane (a clinically relevant concentration) plus 100% oxygen for two hours or a control group that received 100% oxygen for two hours at an identical flow rate in identical anesthetizing chambers as previously described [32], [44]. We chose this anesthesia because the anesthesia with 1.4% isoflurane plus 100% oxygen for two hours is clinically relevant and has been shown to induce caspase activation, increase levels of β-site amyloid precursor protein-cleaving enzyme (BACE) and Aβ, and induce brain inflammation in mice at 5–8 month-old [32], [44]. The anesthesia with 1.4% isoflurane for two hours in mice was employed to demonstrate whether clinically relevant isoflurane anesthesia, which can induce neurotoxicity [44] and neurobehavioral deficits [45] in mice, could also induce tau phosphorylation. We did not use six hours of isoflurane anesthesia in mice because anesthesia for a longer time (e.g., six hours) may increase mortality in mice. We did not measure blood gas and pH values in the current studies because our previous studies had shown that the same anesthesia with 1.4% isoflurane for two hours did not significantly alter the values of blood gas and blood pressure [44]. The mice breathed spontaneously, and the concentrations of isoflurane and oxygen were measured continuously (Datex, Tewksbury, MA). Hypothermia can lead to tau phosphorylation [15], therefore, the temperature of the anesthetizing chamber was controlled to maintain rectal temperature of mice at 37±0.5°C. The anesthesia was terminated by discontinuing isoflurane and placing the animals in a chamber containing 100% oxygen until 20 minutes after the return of their righting reflex. The mice were then returned to their individual home cage until they were humanely killed. Mice were killed by decapitation, and the whole brain tissues were harvested and subjected to Western blot analysis. The numbers of independent experiments in the moue studies were three to six.

We chose AD Tg mice [B6.Cg-Tg (APPswe, PSEN1dE9)85Dbo/J mice] in the experiment to specifically determine the role of Aβ in the effects of isoflurane on the levels of phosphorylated tau, because the AD Tg mice have elevated Aβ levels owing to mutant transgenes for APP (APPswe: KM594/5NL) and presenilin 1 (PS1) (dE9:deletion of exon 9) [35]. We used 5 to 8 month-old mice in the current experiments because our previous studies showed that the isoflurane anesthesia induced caspase activation and Aβ accumulation in the 5 to 8 month-old WT mice [44]. Moreover, the AD transgenic mice used in the experiment usually start to develop elevated Aβ levels during this age range (5–8 month-old) [35]. Finally, both WT and AD Tg mice were age matched in each group.

### Primary Neurons

The protocol was approved by the Massachusetts General Hospital (Boston, Massachusetts) Standing Committee on the Use of Animals in Research and Teaching. Primary neurons from both WT and the AD Tg mice [B6.Cg-Tg (APPswe, PSEN1dE9)85Dbo/J mice] (donating from lab of David Borchelt, McKnight Brain Institute, University of Florida, Gainesville, FL, U.S.A.) were used to assess the potential *in vitro* effects of isoflurane on levels of phosphorylated tau protein. WT and AD Tg mice with a gestation stage of day 15 were killed with carbon dioxide. We then performed a cesarean section to harvest the neurons as previously described [33], [46]. Seven to 10 days after the harvest, the neurons were exposed to isoflurane. The numbers of independent experiments in the neuron studies varied from six to 12.

### Treatment of Neurons

The WT and AD Tg mice primary neurons were treated with 2% isoflurane for six hours under 21% O2 and 5% CO2. We chose this isoflurane treatment because it is clinically relevant and had been shown to induce apoptotic cell death and Aβ accumulation in H4 human neuroglioma cells [26], [27], [42] and in mice primary neurons [32], [33], [47] in our previous studies. The treatment with 2% isoflurane for six hours was employed to demonstrate whether there is potential association of isoflurane-induced caspase activation, Aβ accumulation, and tau phosphorylation in primary neurons. We did not use two hours of isoflurane treatment because treatment with 2% isoflurane for three hours in vitro had been shown not to induce caspase activation [26]. Isoflurane was delivered from an anesthesia machine to a sealed plastic box in a 37°C incubator containing six-well plates seeded with 0.25 million neurons (80% confluent rate) in 1.5 ml culture media per well as described before [33], [46]. The control condition for the isoflurane treatment was 21% O2 plus 5% CO2, which has been shown not to induce cell death or Aβ accumulation [25], [26], [27]. Z-VAD (100 µM) or L-685,458 (0.5 µM) was given to the neurons one hour prior to the isoflurane treatment. The vehicle for Z-VAD and L-685,458 was dimethyl sulfoxide (DMSO), thus DMSO served as control condition of Z-VAD and L-685,458.

### Neuron or Tissue Preparation

The neurons and mouse brain tissues were prepared for Western blot analysis as previously descried [33], [44], [46]. Specifically, primary neurons and mouse brain tissues were homogenized in an immunoprecipitation buffer (10 mM Tris-HCl, pH 7.4, 150 mM NaCl, 2 mM EDTA, 0.5% Nonidet P-40) plus protease inhibitors [(1 µg/ml aprotinin, 1 µg/ml leupeptin, 1 µg/ml pepstatin A) (Roche, Indianapolis, IN)]. The lysates were collected, centrifuged at 13,000 rpm for 15 minutes, and quantified for total proteins with a bicinchoninic acid protein assay kit (Pierce, Iselin, NJ).

### Western Blot Analysis

The Western blot analysis was used to determine the effects of isoflurane on the levels of phosphorylated and total tau protein. The samples were subjected to Western blot analysis as previously described [33], [44], [46]. Briefly, 40 µg (primary neurons) or 60 µg (mouse brain tissues) of each lysate was separated on SDS-PAGE gels and transferred to polyvinylidene difluoride blots (Bio-Rad, Hercules, CA) using a semi-dry electrotransfer system (Amersham Biosciences, San Francisco, CA). The blot was incubated overnight at 4°C with primary antibody, followed by washes and incubation with an appropriate secondary antibody, and visualized with a chemoluminescence system. The levels of phosphorylated tau protein at Serine 262 (Tau-PS262) were recognized by antibody Tau (pS262) (55 kDa, 1∶1,000, Invitrogen, Carlsbad, CA). The total tau was recognized by anti-total tau antibody (55 kDa, 1∶1,000, BD Biosciences, Billerica, MA). Antibodies TAU [pS199] (1∶1,000, Invitrogen, Carlsbad, CA) and TAU [pS422] (1∶1,000, Invitrogen) were used to detect levels of Tau-PS199 and Tau-PS422, respectively. Finally, the antibody to non-targeted protein β-Actin (42 kDa, 1∶5,000, Sigma, St. Louis, MO) was used to control for loading differences in total protein amounts. The signal of the Western blot band was detected using the Molecular Imager VersaDoc MP 5000 System (Bio-Rad Life Science Research, Hercules, CA). The intensity of signals was analyzed by using a Bio-Rad image program (Quantity One, Bio-Rad Life Science Research) or a NIH Image Version 1.37 v (NIH, Bethesda, MD). Brain tissues from Tau knockout mice were used as negative controls in the studies to confirm that the bands in the Western blot following isoflurane treatment represented tau protein.

### Quantification of Aβ Using Sandwich ELISA Assay

Levels of secreted Aβ42 in the conditioned culture media of primary neurons were measured with a Sandwich ELISA assay by using an Aβ42 measurement kit (Invitrogen) as described by Dong et al. [48]. Specifically, 96-well plates were coated with mouse monoclonal antibodies (mAb) specific to Aβ42 (21F12). Following blocking with Block Ace, wells were incubated overnight at 4°C with test samples of conditioned cell culture media, and then an anti-Aβ (α-Aβ-HR1) conjugated to horseradish peroxidase was added. Plates were then developed with TMB reagent and well absorbance was measured at 450 nm. Aβ42 levels in test samples were determined by comparison with the signal from unconditioned media spiked with known quantities of Aβ42.

### Statistics

The changes following isoflurane treatment were presented as percentages of those from the control condition. Data were expressed as mean±S.D. The number of samples varied from three to 12, and the samples were normally distributed. We used a two-tailed t-test to compare the difference between the control condition and isoflurane treatment, and between DMSO and Z-VAD or L-685,458. P-values less than 0.05 (*) and 0.01 (**) were considered statistically significant. The significance testing was two-tailed, and SAS software (Cary, NC) was used to analyze the data.
